# Promoting mental health through a Rural Art Roadshow: perspectives of participating artists

**DOI:** 10.1186/s13033-019-0302-y

**Published:** 2019-06-20

**Authors:** Tony Barnett, Josephine de Deuge, Heather Bridgman

**Affiliations:** Centre for Rural Health, School of Health Sciences, College of Health and Medicine, University of Tasmania, Locked Bag 1322, Launceston, TAS 7250 Australia

**Keywords:** Art, Mental health, Rural health, Community inclusion, Health promotion, Recovery

## Abstract

**Background:**

The therapeutic potential of art to contribute to mental health, well-being and recovery is widely recognised. Benefits include improved self-esteem, self-confidence, communication skills, personal relationships, and fostering greater social inclusion. The *Rural Art Roadshow* is a collaborative art project between the University of Tasmania and not-for-profit mental health and disability support service, Wellways. The *Rural Art Roadshow* is a travelling art exhibition that takes selected artworks submitted by individuals affected by mental illness, to 4–6 small rural towns across Tasmania, Australia. The broad aim of the project is to help reduce stigma and promote a positive image of mental health in rural communities. Whilst the positive impact of art exhibitions has been recognised, there is little research that reports on the experience of participating artists. This study aimed to gain an understanding of the experience of artists impacted by mental illness who participated in the *Rural Art Roadshow*.

**Method:**

A mixed-methods approach was employed. The qualitative data described the experience of 23 artists (17.4% male) who exhibited their work. Data were collected during a series of semi-structured interviews and thematically analysed. This was augmented by survey data (*n* = 145) from visitors to the exhibition over 3 successive years.

**Results:**

Three overarching themes were identified from the interviews: Community Impact, Social Gains and Personal Gains. Sub-themes were: community inclusion, engagement in rural communities, mental health promotion, mental health literacy, connecting with others, enhancing family relationships, creating conversations, positive sense of self, increased self-efficacy and professional recognition for artists. These themes were consistent with visitor survey results.

**Conclusions:**

The findings demonstrate that community art exhibitions can have social and personal benefits for participating artists whilst contributing to rural community wellbeing. This is particularly important for rural communities where isolation and stigma around mental illness is often exacerbated. The *Rural Art Roadshow* is a promising mental health promotion approach for rural and remote areas of Australia. Future research could assess the community health gains of Rural Art Roadshow participation as well as explore the impact on local service providers.

## Background

Australians living in rural and remote areas have poorer health than those who live in metropolitan areas. This includes higher mortality and suicide rates, as well as a higher incidence of chronic disease [1]. Residents of rural areas also have less access to preventative, primary, acute and specialist services, resulting in challenges associated with the diagnosis, treatment and on-going management of mental health conditions. Long travel distances, lack of transport options, costs, long waitlists, and lack of afterhours services [2], are often barriers to accessing timely treatment and support [3–5]. Residents from rural areas can also experience difficulties in accessing mental health services because of the higher levels of stigma associated with mental illness and their social visibility in smaller communities [2, 6]. In order to address some of these issues, alternative programs have been developed for rural dwellers, including those that incorporate art [7].

The benefits of art to mental health have been acknowledged nationally through the Australian National Arts and Health Framework [8]. Participatory arts practices have been shown to promote recovery from mental illness [9]. Recovery in the mental health context is defined as ‘being able to create and live a meaningful and contributing life in a community of choice with or without the presence of mental health issues’ p.2; [10] and has become integral to mental health systems across many countries [11]. The CHIME conceptual framework offers five processes of the recovery journey including Connectedness, Hope, Identity, Meaning and Empowerment [12] and there is evidence that participatory arts impacts all five recovery processes [13]. For people experiencing mental illness, participating in art projects can lead to increased levels of self-efficacy, empowerment, improved well-being, and greater levels of social interaction [14–16]. Art groups can provide a safe, supportive place for reflection, improve self-esteem and self-confidence and have also been credited with facilitating the process of mental health recovery through engaging individuals and increasing social inclusion [17].

Art has also been shown as a means to promote mental health and reduce stigma for art viewers [18, 19]. Koh and Shrimpton [18] found that exhibitions of art by people with experiences of mental illness can successfully promote mental health literacy and contribute to improving community attitudes towards people with mental illness. Publicly hosted mental health themed exhibitions have also been found to create a platform for viewers to reflect on mental health, as well as increase awareness and reduce stigma [20]. For family carers of people who have experienced a mental illness, viewing art in a public gallery is reported to be a positive experience, providing social and psychological support in a safe environment [21].

### The Rural Art Roadshow exhibition

The *Rural Art Roadshow* is a collaborative project between the Centre for Rural Health, University of Tasmania and not-for-profit mental health and disability support service, Wellways. The project aims to develop community resilience, reduce stigma and promote a positive image of mental health in rural Tasmania through a travelling display of art submitted by community members to the Wellways ‘Minds Do Matter’ Exhibition, held in major population centres across Tasmania. ‘Minds Do Matter’ is an annual exhibition where community members affected by mental illness are invited to enter their artwork together with a statement about their work for display. The exhibition aims to promote the therapeutic and reflective process of art and reduce stigma associated with mental illness in the community. From this larger exhibition, and with permission of the artists, approximately 30 art pieces are selected by members of the research team and an art curator, to tour with the *Rural Art Roadshow.* Selection criteria includes the portability of the artwork, subject diversity, and residential location of the artists. For ease of transport, the majority of art work selected are two dimensional such as paintings or textile works that can be hung, with a small selection of pre-assembled three dimensional pieces such as sculptures or wood carvings.

The *Rural Art Roadshow* travels over 4 weeks, visiting between four and six rural communities each year. Communities are chosen because of their relative isolation and disadvantage in accessing mental health promotion services. Eight different towns have been visited over the 3 years the *Rural Art Roadshow* was established (Table 1), with populations varying from 316 to 4347 residents [22]. All communities are classified between 3 and 4 on the Australian Standard Geographical Classification-Remoteness Area (ASGC-RA) (1-Major cities of Australia to 5-Very Remote), and between 5 and 6 on the Modified Monash Model (MMM) in terms of remoteness [23].

**Table 1:** Communities visited by the *Rural Art Roadshow* 2015–2017

Towns	Population [15]	ASGC-RA [16]	Modified Monash Model [16]	Year visited		
				2015	2016	2017
Dunalley	316	3	5		X	
Fingal	336	3	5		X	X
George Town	4347	3	5	X	X	X
Queenstown	1755	4	6	X		
Scottsdale	2373	3	5	X	X	
Sheffield	1552	3	5		X	
Smithton	3881	3	5	X	X	X
St Helens	1449	3	5			X

*ASGC-RA* Australian standard geographical classification-remoteness area

An event management plan, tailored to each individual community was developed by the research team to deliver the *Rural Art Roadshow*. The plan included details for securing an accessible exhibition space, strategies for engagement with local community groups and stakeholders, event publicity, catering for each exhibition opening and logistics for secure transport, packing and unpacking of the art in order to mitigate the risk of loss or damage.

To facilitate maximum exposure for the art and to increase the promotion of mental health, the exhibition was held in locations well utilised by the community such as local cafes or community spaces, such as child and family health centres or neighbourhood houses. An opening event was held in each community, with local government representatives, service providers, key community stakeholders, and community members invited to attend. Entry was free, and the exhibit usually remained on display for 1 week. At each opening, local artists whose work had been included in the *Rural Art Roadshow* were invited to talk about their experience as an artist and as a person affected by mental illness. This aimed to assist understanding and to promote a more positive image of mental health and well-being.

Whilst some studies have identified the positive benefits of exhibitions in promoting mental health and wellbeing to exhibition visitors [20, 24], few studies have examined the experience of artists participating in a travelling art exhibition in the rural context. This study aimed to gain an understanding of the experience of artists impacted by mental illness who had participated in the *Rural Art Roadshow* at one or more exhibitions over the 3-year period.

## Methods

A mixed-methods approach was employed that included a short survey made available to visitors at each exhibition as well as semi-structured interviews with artists about their experience. Mixed methods approaches can address some research questions more comprehensively as compared to use of qualitative or quantitative methods alone [25].

### Visitor survey

The *Rural Art Roadshow* was evaluated through the use of a short anonymous survey made available at each exhibition for community members and artists to complete and put into a sealed drop box. The same survey tool was used over the 3 years and listed six statements to which visitors were asked to indicate their level of agreement on a five point scale (strongly agree to strongly disagree). Two open-ended questions ‘what is something you have learned from the roadshow?’ and ‘how could the roadshow be improved?’ allowed visitors to provide additional comments.

Numeric survey data were analysed using IBM SPSS Statistics Version 24. More favorable responses on the survey were indicated by higher scores. The internal consistency for the instrument for the sample was high (Cronbach’s Alpha = 0.93). Written comments were collated and subject to simple content analysis [26].

### Interviews

All artists who had contributed an art work/s to the *Rural Art Roadshow* between 2015 and 2017 were invited to participate in the study. Inclusion criteria were being able to read and speak English and over 18 years of age. Artist participants were recruited through a mail out, that was followed up with a phone call. This ensured that the opportunity to participate was offered to all artists who may have changed their address, and to ensure there was a mix in gender, type of mental illness experienced, carer status, and geographical location. This allowed for information-rich data to be collected with a small sample size [27]. The research team aimed for a sample size of 20 participants. This is considered reasonable as a sample size for this type of qualitative research [28] as it has been suggested that little ‘new’ information is discovered after sampling this number of people [29] and is consistent with previous literature [15]. Of the 58 artists contacted, 23 (40%) agreed to participate.

### Procedure

Interviews with artists were conducted by one member of the research team either over the phone or in person at the University of Tasmania between July 2017 and February 2018. Artists were sent the information sheet and consent form prior to interviews. The interview guide (Table 2) included questions about artist experiences of participation in the *Rural Art Roadshow* and the role of art in the participant’s life. Interviews ranged from 10 to 50 min in duration. Field notes were taken during each interview and referred to during subsequent data analysis. These notes provided additional context to the study. Artists were invited to review their interview transcripts and to make any modifications they felt were needed with a 2-week turnaround request.

**Table 2:** Artist interview guide

Questions
Could you tell me a about your experience participating in the *Rural Art Roadshow*?
Did you attend an opening? How did you find this experience?
What has it been like for you to talk about your art with community members at the *Roadshow*?
Would you recommend this experience for other artists showing their works in the *Rural Art Roadshow*? If so why?
Is there anything you can think of that would improve the event? For yourself? For others?
Can you tell me about some of the roles art has had in your life?

Interviews were audio-recorded and transcribed verbatim into Microsoft Word and then cross-checked by a member of the research team against the audio recording for errors and omissions. To maintain confidentiality, participants were assigned a numerical code in each transcript. Narrative data were then imported into QSR-Nvivo v10.0 software [30] and analysed using inductive thematic analysis [31] to identify patterns and recurring themes.

One member of the research team initially coded all transcripts by identifying the same word, synonym, phrase or idea embedded within each text. These were then checked by the other members of the research team and discussed until a consensus was reached on what to name each code. Codes were then categorized based on their similarities and relationship to each other within each transcript and emergent themes then developed from these groupings. The results were compared and discussed at regular meetings by the full research team until no new themes were generated and agreement was reached [32].

### Ethics approval

The Human Research Ethics Committee of Tasmania (H0015338) granted approval for the study.

## Results

### Quantitative results

There was little variance between survey responses to items from visitors to the *Rural Art* *Roadshow* across the 3 years (*n* = 145). Overall results showed there was a strong level of agreement that the exhibition was a welcome addition to the community and that they would encourage others to see it (Table 3). Community members also believed that the *Rural Art Roadshow* would help to promote conversations about mental health and reduce stigma in the community. Responses for each year were relatively low ranging from 43 to 54. Close to half the responses also contained written comments, with the majority providing positive feedback to what they had learned from the exhibition including the relationship between art and mental health and the importance of community discussion in promoting mental health awareness. Respondents also provided suggestions to improve the roadshow that included increased publicity, a broader range of artwork on display and more opportunities for discussion with artists (Table 4).

**Table 3:** Visitor evaluation of the *Rural Art Roadshow* (n = 145)

Questions	Mean (SD)	Median
The *Rural Roadshow* I have viewed…		
Was helpful to my understanding of mental health	3.96^a^ (0.84)	4
Was a welcome addition to our community activities	4.51 (0.70)	5
Is something I believe will help promote conversation about mental health	4.27 (0.74)	5
Is something that should be repeated each year	4.39 (0.70)	5
Will help lessen stigma of mental health in our community	4.38 (0.76)	5
Is something that I would encourage others to see	4.56 (0.67)	5

^a^Likert Scale of 1–5 with 5 indicating strong agreement

**Table 4:** Top four visitor learnings from and improvement suggestions for the *Rural Art Roadshow*

What is something you learned from the Roadshow? (responses in descending order of frequency)
The relationship between art and mental health
The importance of community discussion and promoting mental health awareness
Talent of the local community
Different ways Mental Health can be displayed through Art
Suggestions for improvement
Increase publicity about the Roadshow
Offer a broader range of artwork
Create more opportunities for artist involvement
Hold art workshops in association with the exhibitions

### Qualitative results

**Fig. 1:**
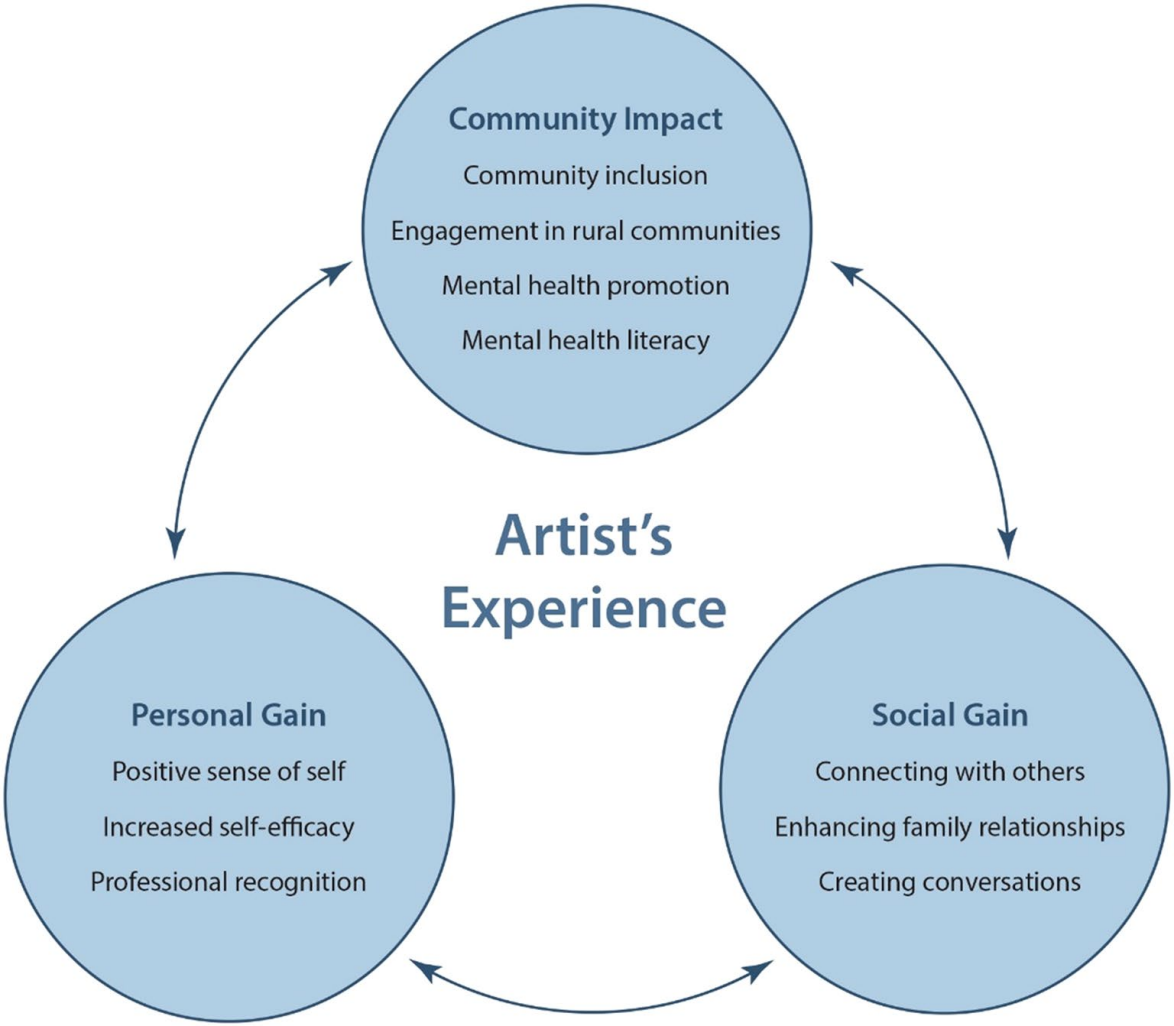
Qualitative themes of the Artist’s Experience of the *Rural Art Roadshow*

Twenty-three individual interviews (17.4% males) were conducted with artists. Two had been involved in the *Rural Art Roadshow* in 2015, 11 in 2016 and 10 in 2017. Of the 23 artists, seven (30%) had attended a *Rural Art Roadshow* opening. The art experience of the artists ranged from entrants who had not previously exhibited, through to artists that had showed work on multiple occasions. Three participants reported being carers for a family member with a mental illness, eight disclosed a mental health condition and 12 reported being ‘affected’ by a mental illness. Ten sub-themes were identified from the transcripts. These were then grouped into one of three major themes: community impact, social gain, and personal gain. A number of sub-themes related to more than one theme. For example, ‘creating conversations’ was associated with both the community impact and social gain. Connections and hypothesized relationships between themes are represented in Fig. 1.

### Community impact

Artists discussed how they believed the *Rural Art Roadshow* had an impact on the community and this was represented by four sub-themes: community inclusion, engagement in rural communities, mental health promotion and mental health literacy.

#### Community inclusion

According to artists, the exhibitions were well attended, as an inclusive environment for all community members, with openings attended by between 15 and 60 people. Artists also discussed how the event provided an opportunity for individuals who may have felt socially isolated to come into a safe community space and engage with others:

*And I felt that it drew a really good cross section of the community… it was really nice to be in such mixed company all celebrating something positive together (Participant 1)*

*People don’t go out of their way to look for things…. whereas if it’s things they can attend, they can connect with other people because it’s the connection that’s so important (Participant 23)*

#### Engagement in rural communities

Many artists praised the event as a way for them to engage with rural communities. The engagement was viewed as a gain for the community as it provided members with an opportunity to connect with artwork that was not usually available to them:

*‘cause we’re pretty rural in Tasmania so it’s awesome that those people have avenues to connect with the art community and other artists as well (Participant 15)*

*I think going to smaller communities… I thought that was great (Participant 18)*

#### Mental health promotion

Artists discussed how the *Rural Art Roadshow* could help change public perceptions of mental illness as well as raising awareness of carers and helped to reduce the stigma often associated with mental illness:

*Because in [town] they were showing in a bakery shop… which I think is really good because that’s really bringing the issue of mental illness to a wide variety of people and I was pleased about that …. many more people are going to see it and have it there and be asking others or chatting to the people and asking about the art and perhaps talking about their own personal experience or experiences of people that they know (Participant 10)*

*The Rural Art Roadshow just as a way to, not just help people express themselves, but also for the viewers to be able to maybe understand a bit more. Hopefully it breaks down stigmas and helps with the understanding better of mental illness and the challenges (Participant 17)*

*Highlights two different sides and it also draws attention in the community that this is going on and there are very productive ways of dealing with the illness or the moods or whatever… Both sides of the coin: the family that’s impacted through no fault of the person with the illness, and to the people who have to live with it (Participant 7)*

#### Mental health literacy

Artists discussed how the *Rural Art Roadshow* allowed them to increase their knowledge of mental health issues and services. This occurred by artists engaging with service providers that attended the *Rural Art Roadshow* and others who also had a mental health illness, or were caring for someone with mental health challenges:

*…there was one chap who was obviously, I can’t think what you’d call him, to do with mental health and he helps people and I was interested in talking to him, and that he was going around talking to other people (Participant 19)*

*Just knowing about the art show actually helped me to learn more about my illness and develop skills around managing it. But I’ve also been in the opportunity where I’ve been able to gain knowledge and information (Participant 1)*

### Social gain

The Social gain theme was characterised by three sub-themes: connecting with others, enhancing family relationships and creating conversations.

#### Connecting with others

Artists spoke positively about the connections they were able to make with other community members and artists:

*Just getting yourself out there and mixing with other artists that you wouldn’t normally mix with and other people that you wouldn’t… some of the people that were there were just from the community (Participant 23)*

#### Enhancing family relationships

Artists discussed how the *Rural Art Roadshow* allowed them to engage with family through providing a platform to demonstrate support, as well as a bonding experience through the creative process prior to the exhibition:

*My eldest daughter, she goes to Wellways and she has had a long, many, many years battle with depression and one of the things that we have in common is our art. So she asked me if I would like to put anything in the exhibition. So it was something that shows my support for her but also something that we like doing together (Participant 21)*

*But if you saw our living room you’d see that creativity doesn’t stop. So just yesterday my son was out there cutting some wood, he was making some props for a movie that he’s going to shoot with some of his mates. And my daughter is creating little sculptures on the table and my wife’s abstract art is up against the fireplace that she’s someway through. You know what I mean, so the family that creates together stays together (Participant 5)*

#### Creating conversations

Artists also agreed that the *Rural Art Roadshow* helped to create conversations about mental health. Artists recounted how individuals had shared their stories and experiences and that they felt this had a positive impact at both community and individual level:

*There were good conversations about mental illness there and a number of people shared with me on that occasion that they also had a lived experience of mental illness…. but it certainly opened conversations in the community (Participant 1)*

*I spoke to some ladies and gentlemen. I spoke to different people. Someone raised the aspect of depression on one of the paintings and she started talking about what that meant to her and just started talking about mental health and mental illness and she started opening up about her experiences with it. And I think that’s part of the whole beauty of the Rural Art Roadshow is that it creates discussions about mental health and recovery (Participant 2)*

### Personal gain

The personal gain theme consisted of three sub-themes: positive sense of self, increased self-efficacy and professional recognition.

#### Positive sense of self

Artists commented that the *Rural Art Roadshow* gave them a sense of pride and achievement, particularly because their art had been selected out of a large group of artworks for display around the state:

*Obviously it was a positive experience for me to think that the art was selected in a small group of art that was going to travel the State and that other people would see it and hopefully be encouraged by it (Participant 5)*

*For me, that was a personal achievement that I could actually accomplish something (Participant 3)*

*Every step of the way it’s been very supportive, encouraging and it’s been good for me because it just gives me some introspection and some time to just reflect on all that I’ve been a part of, that I’ve seen, heard, and its on*-*going (Participant 16)*

#### Increased self-efficacy

Artists discussed how the *Rural Art Roadshow* improved their confidence and their belief in their art ability as well as their ability to discuss their mental health. One participant reported that they had at times not spoken about their mental health because they felt ashamed of their mental illness, however, the *Rural Art Roadshow* provided them with an opportunity to speak out and feel empowered:

*It’s something that has given me an opportunity to talk in public. There’s been a lot of secrecy around my mental illness. My family of origin are still in denial about it so for me to be able to speak out about it has been really empowering… So through the Roadshow I feel I’ve been given a voice and been able to talk about issues around mental illness. (Participant 1)*

*It’s like a bit of a confidence boost seeing how much you have improved. And being acknowledged (Participant 12)*

#### Professional recognition

A number of artists felt that the *Rural Art Roadshow* was a professional step as it provided them with an opportunity to exhibit their work publicly and receive feedback from other artists as well as the community more broadly. It also allowed them to gain additional exposure following the larger Wellways exhibition in a major population centre:

*Just having the experience of exhibiting and being able to share your work with other people and not anybody, not necessarily being a recognised artist or thinking of yourself perhaps that way but just people who draw can share in that whole experience. (Participant 18)*

*I think the more exposure to people’s art, especially the people who may not be recognised artists as such but just general community (Participant 17).*

*It’s a nice way to get exposure when you’re just starting out (Participant 18)*

### Opportunities for improvement

Artists provided a number of suggestions for improvement. These included coaching around the artist statement that accompanied each piece of artwork. Artists suggested that having awards at the *Rural Art Roadshow*, would add to the excitement of the exhibition. They suggested that holding art workshops in association with each exhibition may also strengthen engagement with communities and allow connections to be made with others in the community experiencing a mental illness.

A few artists expressed limited awareness of the role of the *Rural Art Roadshow* and suggested additional promotion and an online presence would allow others who were unable to get to the exhibition, or who may be from other rural towns, the opportunity to view the artwork. Using social media to display and advertise the art would also increase publicity and exposure of the event:

*I think someone took photos last year but even if someone, just on their iPhone, took a video of the space with the artwork in it or something like that and put it online that would be fantastic. So at least people could somewhat experience the exhibition without necessarily having to go there themselves…. It just means that for my family that are on the mainland I can send them links and they can see what we have been involved with here (Participant 5)*

Another recommendation made by artists was increased collaboration with community groups and organisations such as schools and health services. Additional collaboration would increase the ownership in communities and assist the exhibition in becoming sustainable as well as bringing more people to the exhibition and being able to offer more locally tailored information about support and services available.

## Discussion

This study examined the experiences of artists who participated in the *Rural Art Roadshow* and perspectives of community members who attended the exhibit. There was evidence that the exhibit made an impact on community members and that there were social and personal gains for the participating artists.

The analysis of 145 surveys, offered evidence that the *Rural Art Roadshow* had a positive impact on community members and visitors. Responses from the survey suggested that the exhibition could be used as a community tool to reduce stigma and promote discussions about mental health, whilst also showing the beneficial relationship between art and mental health and well-being. These results on community impact were further supported through the artist’s interviews. These findings are consistent with previous research that has shown that public art displays can increase mental health awareness [20], empathy and understanding of mental illness [18–20] and as well as an appreciation of the creativity of those with a mental illness [18]. The artists viewed the *Rural Art Roadshow* as a positive event that helped create an inclusive community environment for promoting mental health and mental health literacy.

Artists also highlighted the social and personal gains that they had experienced from their participation in the road show, including an increased positive sense of self and self-efficacy, as well as developing social connections and creating conversations. These findings align with those from previous research into the role of participatory arts and the CHIME recovery framework where connectedness has been the most frequently mentioned recovery process contributing to social inclusion [9, 13]. Participating in a community art project also increases participants social contact and inclusion [33], which is an important component of mental health recovery [14, 34]. In line with previous research, there was also evidence that contributing and participating in community arts projects, including exhibitions, improved artists self-confidence and empowerment through improving self-efficacy and self-worth [17, 35]. Working towards a specific event, such as the *Rural Art Roadshow,* contributes to optimism and pride in achievement [9].

Beyond the personal and social gains of the artists, the outcomes add to the wider literature on participatory arts and mental health demonstrating the benefits of a travelling rural art exhibition to the broader community. Stickley et al. [13] identified several common features associated with the successful promotion of mental health outcomes in participatory arts activities to promote social inclusion. These included: delivery in local communities; attempting to be non-stigmatizing; flexibility; promoting social engagement; involvement of exhibitions; and delivery within a specific period of time. Consistent with these features, the *Rural Art Roadshow* offers a potential template for promoting positive mental health outcomes in the rural context.

Quality improvement suggestions include identifying other ways to disseminate the art, such as the use of social media for online exhibitions and discussion boards to promote the benefits of art and mental health. The use of online mediums, including websites and social media sites such as Facebook to create virtual art exhibitions has been widely used [36] and can be beneficial by increasing the reach and accessibility of a display. Research investigating viewers experiences of attending a virtual gallery online, found that they had positive responses to the digitalization of the exhibition, though this innovation would not necessarily replace the experience of visiting a gallery in person [37].

Another suggestion for the *Rural Art Roadshow* included further engagement and collaboration with artists, local support groups, community organisations, and activities to increase community ownership. This could potentially facilitate the sustainability of the *Rural Art Roadshow* through drawing on local mental health advocates and community resources to assist with organising, publicising and running the event. Health gains of the community should also be investigated in future research as well as the impact on local health service providers.

### Limitations of the study

There were a number of factors that may have influenced this research and should be considered within the overall context of the study. The quantitative aspect of the study relied on community members to self-report their individual perceptions of what they gained from a visit to the *Rural Art Roadshow*. Survey responses should be considered against the potential effect of acquiescence or social desirability response bias [38]. Those visitors that did complete the survey and return it via the drop box may have been more positively disposed to the project and what it was trying to achieve. The survey data may have also been more favourable from the communities where the *Rural Art Roadshow* had been to multiple times.

The timing of the interviews was also more favourable for those who attended the 2017 *Rural Art Roadshow*, as interviews were conducted 1 month after the show, compared to the 2016 interviews which were conducted 8 months post show and the 2015 artists who were interviewed 21 months post show. History and recall ability could have impacted on artists reports, however the outcomes were reasonably consistent across the sample despite the time lag differences.

There is evidence that some groups are under-represented in mental health research [39] due to reasons including stigma attached to mental illness, distrust and/or fear of research and severity of illness. This may indicate that those who are more affected by their mental illness may have refrained from submitting their work to the Wellways art show or may have been reluctant to participate in the *Rural Art Roadshow* because of fear they may have been identified by their local community when the work was exhibited. These factors may affect the generalisability of the study findings.

While the study captured the perspectives of participating artists and community members that attended the *Rural Art Roadshow,* feedback was not obtained from other important stakeholders such as family members or carers of the artists affected by mental illness, local health service providers, community groups, the education sector or the broader community. Future research could focus on exploring the impact of the *Rural Art Roadshow* from these perspectives.

### Recommendations

This study identified a number benefits of the *Rural Art Roadshow* to rural communities and to participating artists and some areas for improvement. It is recommended that future iterations focus on early and consistent community engagement, connecting with regional arts groups and key community champions to encourage higher levels of local input. Future *Roadshows* should also include a promotion and publicity plan tailored to each community to ensure the exhibition and associated mental health promotion message is widely disseminated. Promoting the *Roadshow* to the participating artists themselves is also recommended to strengthen the message around inclusion and community acceptance. Finally, the potential for using technology, for example online ‘exhibition spaces’ or connecting artists with communities virtually should be considered.

## Conclusion

Findings from this study contribute to the limited evidence concerning the personal and social benefits of participating in an art exhibition for artists impacted by mental illness. Findings extend the current literature by demonstrating the community benefits that can arise from a travelling art roadshow in a rural setting, where there is often isolation and increased stigma around mental health. Future research could assess the community health gains of participation as well as the impacts on local health service providers. The *Rural Art Roadshow* is a unique and promising model for fostering conversations about mental health and promoting community inclusion in rural and remote settings.

## Data Availability

In order to maintain confidentiality of participants the raw data is not available publicly. Please contact the corresponding author for further information.
